# Description of four cases of male genital schistosomiasis (MGS) in children and adolescents, with a scoping review

**DOI:** 10.1017/S0031182025000241

**Published:** 2025-12

**Authors:** Joachim Richter, Sekeleghe A. Kayuni, J. Russell Stothard, Charles Émile Ramarokoto, Andreas K. Lindner, Daniela Fusco, Hermann Feldmeier, Amaya L. Bustinduy, Jennifer F. Friedman

**Affiliations:** 1 Institute of International Health, Global Health Center, Charité Universitätsmedizin, Corporate Member of Free University and Humboldt University and Berlin Institute of Health, Berlin, Germany; 2 Centre for Tropical and Travel Medicine, Swiss Tropical and Public Health Institute, Basel, Switzerland; 3 Department of Tropical Disease Biology, Liverpool School of Tropical Medicine, Liverpool, UK; 4 Epidemiology Unit, Institut Pasteur, Antananarivo, Madagascar; 5 Implementation Research Group, Bernhard Nocht Institute for Tropical Medicine, Hamburg, Germany; 6 Institute of Microbiology, Charité Universitätsmedizin, Corporate Member of Free University and Humboldt University and Berlin Institute of Health, Berlin, Germany; 7 Department of Clinical Research, Faculty of Infectious and Tropical Diseases, London School of Hygiene and Tropical Medicine, London, UK; 8 Lifespan Center for International Health Research (CIHR), Department of Pediatrics, Warren Alpert Medical School of Brown University, Providence, RI, USA

**Keywords:** adolescents, children, hydrocele, hypogonadism, male genital schistosomiasis, minors, *Schistosoma*, schistosomiasis, testicular masses, ultrasound

## Abstract

We present four cases of male genital schistosomiasis (MGS) within children and adolescents opportunistically encountered as part of a wider screening programme for imported schistosomiasis in Germany and community outreach screening in Mali. Such genital manifestations in young children and adolescents are often overlooked but can include hydrocele, hypogonadism, varicocele, cutaneous granulomata on the penis and scrotum, echogenic spots in the prostate and the epididymis, alongside testicular masses. Though these cases appear sporadic, from our scoping literature review, they draw fresh attention on MGS in young children and highlight wider confusion with other congenital, neoplastic and infectious disease. These might include an insufficient closure of the tunica vaginalis, malignancies or lymphatic filariasis. Frequently haematuria is not present. One typical sign indicating MGS in adults, i.e. haematospermia is not present before puberty. Another reason of missing MGS cases may be that screening with scrotal or transabdominal ultrasonography are not easily accepted unless the reason for it is not extensively explained beforehand and that transabdominal ultrasonography is less sensitive for revealing prostatic lesions than transrectal ultrasonography.

## Graphical abstract

**Figure:**
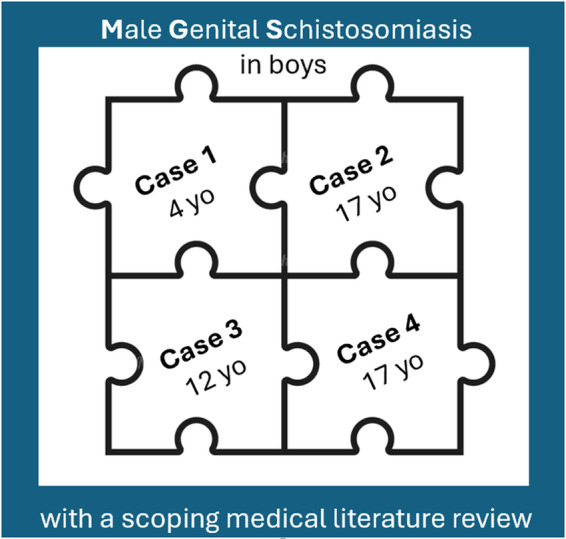

## Introduction

Schistosomiasis is a neglected parasitic infection caused by blood fluke trematodes of the genus *Schistosoma*. Four out of six *Schistosoma* species pathogenic to humans have been described as being capable of inducing disease sequelae within the genital tract: *S. haematobium*; causing urogenital schistosomiasis, *S. mansoni, S. intercalatum*, and *S. japonicum*; mainly causing intestinal and hepatosplenic schistosomiasis (Lee et al., 2000; Shekhar et al., 2000; Yu et al., 2013; Bustinduy et al., 2023). Recently, the zoonotic species *S. matthei* has also been found to cause human genital schistosomiasis (Kayuni et al.,^2024^). Globally, approximately 240 million people are infected (about 90% living in sub-Saharan Africa) (WHO 2020). Health complications arising from genital schistosomiasis have been more and more acknowledged, especially those occurring in women (Abul Kahir et al. 1980; Richter et al., 1995, 1996, 2008; Kjetland et al., 1996; Helling Giese et al., 1996a; Helling-Giese et al., 1996b; Poggensee et al., 1998; Schanz et al., 2010; Christinet et al., 2016; Kayuni et al. 2020; Kayuni et al., 2024; World Health Organization, 2020; Bustinduy et al., 2022; Fusco et al., 2022; Kutz et al., 2023; Shanaube et al., 2024). However, little is known about the frequency of genital manifestations of schistosomiasis in younger children and adolescents, particularly in males. To shed light on the latter, we report on four cases of MGS in male children and adolescents, and place these cases within a wider appraisal of available literature.

## Ethical considerations

Mandatory routine infectious and parasitic diseases screening of unaccompanied minor refugees (UMR) arriving in Berlin is performed at the Institute of Tropical Medicine and International Health of Charité – Universitätsmedizin Berlin, and previous findings have been described in detail elsewhere (Theuring et al., 2016).

## Case reports

### Case 1

**Figure 1.**
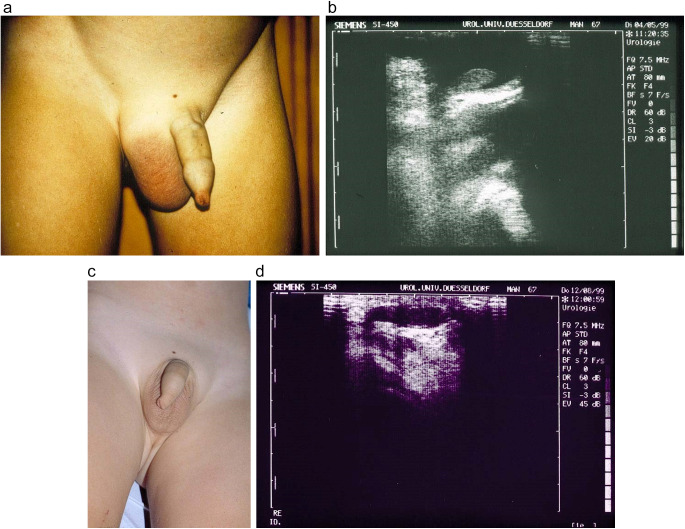
Two-year-old German boy with *Schistosoma mansoni* infection, who had grown up in Kenya and Tanzania. Scrotal swelling and ultrasonography showing hydrocele before (1a, 1b) and six weeks after praziquantel (1c, 1d).

A 4-year-old boy, son of German volunteers living in Tanzania for 2 years, had intermittent non-febrile bloody diarrhoea for 5 months. After returning to Germany he was examined and presented with a painless swelling of the scrotum and livid skin discoloration, with congestion of the dorsal vein of the penis, accompanied by tenderness and swelling of two inguinal lymph nodes. At interview the parents recalled that he used to play near a river in Tanzania; they remembered no trauma. The diagnosis of schistosomiasis was confirmed by faecal microscopy, where viable ova of *S. mansoni* were found. Urinary sediment was normal; *Schistosoma* ova were not detected by microscopy of urine collected over 24 h with the whole volume filtered through microfilters (Nucleopore, Corning, Acton, USA). A differential white blood cell count showed eosinophilia (10%, 600 µL^−1^). Serology for anti-*Schistosoma* antibodies (anti-adult worm antibodies, an immune-haemagglutination assay) was highly positive. Amoebiasis, filariasis, toxocariasis and urogenital tuberculosis were excluded by clinical, parasitological, serological and ultrasonography means and antigen testing for *Wuchereria bancrofti* (Amaral et al., 1994, Chung et al., 1997, Weil et al., 1997). On abdominal ultrasonography, he had nonspecific hepatosplenomegaly, as encountered in the early stages of the disease (Barata et al., 1999) but without typical signs of schistosomal hepatic fibrosis (Richter et al., 1992). No abnormalities of the urinary tract were detected (Niamey Working Group, 2000). Scrotal ultrasonography showed an echo-free fluid in the scrotum; the testis, epididymis, prostate and seminal vesicles were normal (Figure 1). A consultant paediatric urologist interpreted these findings as due to a patent processus vaginalis and recommended surgical division of the processus. However, more invasive investigations were declined by the boy’s parents because we suspected a causal relation between schistosomiasis and the boy’s hydrocele. He was treated with one standard dose of praziquantel at 40 mg kg^−1^ and surgery was postponed. The treatment was repeated at the same dose over 2 days 8 weeks later, owing to the detection of viable ova still being excreted in the stool; 16 weeks after therapy *Schistosoma* ova were absent in the faeces. The hydrocele had disappeared and remained so during the following 12 months, when the boy was last reviewed.

### Case 2

**Figure 2.**
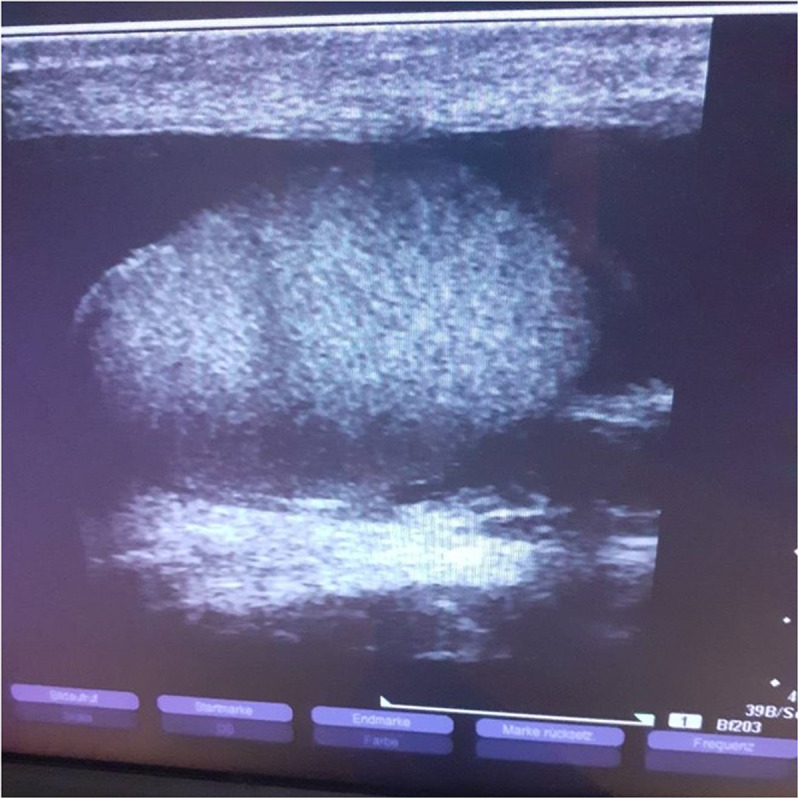
Scrotal ultrasonography of a 17-year-old Fulani refugee from Guinea Conakry with *Schistosoma mansoni* infection. Right testis immerged in anechoic scrotal fluid.

Unaccompanied minor refugees are routinely screened in our outpatient clinic. Here, we attended a 17-year-old male Fulani adolescent from Sierra Leone. He had been treated successfully for disseminated tuberculosis a year earlier. At his presentation in our OPD, his differential blood count revealed an eosinophilia of 1.1 nl^−1^ (norm < 0.5) and IgE was increased to 4646 ku l^−1^ (norm < 100). Serology revealed positive antibodies against filariae, *Strongyloides stercoralis* and schistosomes. Microscopy of microfiltered urine collected during 24 h did not reveal the presence of helminths or their ova. Microscopy of four skin snips and of anticoagulated full blood microfiltered through a nuclepore filter at lunchtime and after provocation with di-ethyl-carbamazine were all negative. Microscopy of enriched fresh stool samples for helminth larvae according to the Baermann method was negative for worm larvae but instead, ova of *S. mansoni* were found. The patient was initially seen by a female doctor. At the second visit he asked for being seen by a male doctor because he was ashamed to report a recurrent scrotal swelling with disappearance of his penis inside the swollen scrotum. Ultrasonography of the scrotum revealed a hydrocele of the right scrotum and to a lower degree of the left side (Figure 2). Adult filariae (‘filarial dance sign’) were not detected in the scrotal lymphatics (Al-Saeed et al., 1994). Therapy with ivermectin 200 μg kg^−1^ was given and repeated after two weeks followed by praziquantel three doses of 40 mg kg^−1^ body weight. Since during the 12 months following antiparasitic therapy the hydrocele persisted, surgical therapy was performed in order to completely cure his hydrocele.

We cannot exclude that the aetiology of hydrocele in his case was multifactorial. He had been cured in the past from disseminated tuberculosis and had probably been previously infected by filariae as proven by positive antibodies against filariae. The moment he was attended in our service, he suffered from active schistosomiasis. Anti-helminthic alone was not sufficient for curing his hydrocele, so that we opted for additional surgical therapy which was finally successful. The patient was well until three years later, when he was last reviewed.

### Case 3

**Figure 3.**
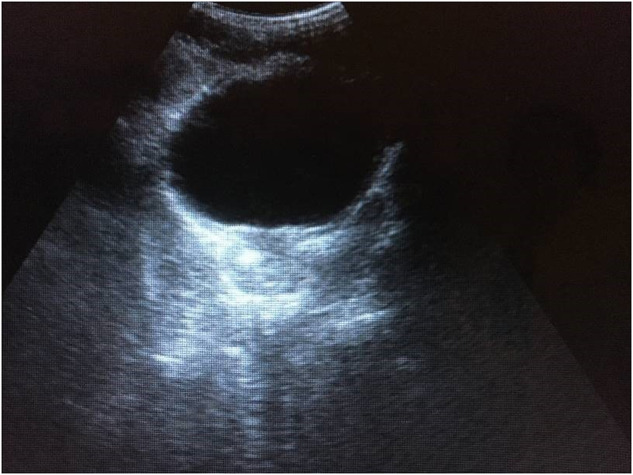
Hyperechoic spot in the prostate of a 12-year-old boy from Mali with *S. haematobium* infection.

During a training course on ultrasonography in urinary schistosomiasis in Mali 25 children were selected, 13 of whom (52%) excreted ova of *S. haematobium* in urine. Among the children one 12-year-old boy was identified by ultrasonography who presented a hyperechogenic spot measuring 7× .4 × 4 mm in the right prostate lobe (Figure 3). Microfiltration of noon urine revealed a quantitative egg excretion of 11 eggs/10 ml urine. Findings of seminal vesicles, urethers and kidneys were unremarkable. The patient was treated by praziquantel with a single standard dose of 40 mg kg^−1^ body weight. The patient was lost to follow-up.

### Case 4

This case was seen among other minor refugees to Germany from Guinée Conakry, presenting with a hydrocele which reversed after three doses of praziquantel at 40 mg kg^−1^. Details are described in Table 1.

**Table 1.** Reports on genital schistosomiasis in male children and adolescents

First author/year	Country of infection	No. of patients	Age. of patients (years)	*Schistosoma species*	Disease manifestation	Clinical suspicion/accidental finding?	Haematuria	Urine microscopy for ova	Diagnosis by	Therapy	Outcome
Richter *et al*./2002	Tanzania	1	4	*S.m.*	Hydrocele, tender swelling of inguinal lymphnodes, congestion of vena dorsalis penis, hepatosplenomegaly	Processus vaginalis/y	No	Neg	Ova in stool		
	PZQ 40 mg kg^−1^, 3 doses	Complete recovery after 6 weeks									
Ze Ondo *et al*./2014	Senegal	1	6	*S.h.*	Hard local swelling (granulomatous mass) of the testicle and small hypopigmented lesion on the scrotum	Tumour/y	No	Neg	Histology	Excision, PZQ 40 mg kg^−1^	Complete recovery
Lukacz *et al*./1989	Hungary ex Egypt´Sudan?	1	7	*S,h,*	Testicular tumour mimicking malignancy	Malignancy/y	No	NA	Histology	Excision	Not reported
Gelfand *et al*./1940	Zimbabwe	1	8	*S.h.*	Left testicular swelling (schistosomal orchitis with hydrocele)	Tumour/y	No	Not done	Histology	Surgery	Not reported
Rambau *et al*./2011	Tanzania	1	9	*S.h.*	Painful scrotal swelling, testicular atrophy. hydrocele, thickened						
tunica vaginalis and small nodules on the surface of the testis	/y	No	Not done	Histology	Orchiectomy, PZQ 40 mg kg^−1^						
Urine microscopy not done	Not reported										
Oguntunde *et al*./2020	Nigeria	1	9	*.S.h.*	Hydrocele thickened spermatic chord	/y	No	Not done	Histology	Surgery, followed by PZQ 400 mg.	on Observation
Joshi/1967	Sierra Leone	1	10	*S.h. + S.m.*	Granulomatous infarction of testicle	Malignancy/y	No	Not done	Histology	Not reported	Orchiectomy because of suspected cancer
Adeyemi Doro *et al*./1979	Nigeria	1	10	*S.h.*	Papular exanthema of the perineal skin	STD?/y	No	Not done			
(schistosomiasis not expected)	Histology	Niridazole 2 courses of 100 mg/thrice daily for a week	cure								
Eltayeb *et al*./1969	Sudan	1	10	*S,h,*	Swelling of the right testis	Tumour/y	No	Urine and stool microscopy neg	Histology		
(epididymits)	Orchectomy of the right testis	Cure									
Madden/1911	Egypt	1	11	*S.h.?*	Scrotal swelling	Tumour/y	Na	NA	Histology?	Not reported	Not reported
Pawel *et al*./2008	Liberia	1	11	*S.m.*	Inguinal hernia with hydrocele	Hernia/y	No	ND	Histology	Herniotomyy	
Monnet *et al*./1972	Tunisia	1	12	*S.h.*	Swelling of the left testicle	Tumour?	No	Not done	Histology		
Ihekwaba *et al*./1992	Nigeria	1	12	*S.h.*	Scrotal swelling	Tumour/y	No	NA	Histology	NA	NA
Ramarakoto unpublished	Mali	1	12	*S.h.*	Hyperechogenic prostata spot	Screening/y	Na	Pos	Urine microscopy	PZQ 40 mg kg^−1^	Lost to follow-up
Aminu *et al*./2023	Nigeria	1	12	*S.h.*	Swelling and papula of the skin of the left hemiscrotum	Tumour?/y	Yes	Not done	Histology	PZQ 40 mg kg^−1^	Improvement after PZQ
Ekenze *et al*./2015	Nigeria	1	13	*S,h,*	Huge scrotal swelling with a granulomatous mass, swelling of inguinal lymphnodes	Malignancy/y	Yes	Neg	Histology	Excision, followed by PZQ 400 mg	Not reported
Githae/1992	South Africa	1	13	*S,h,*	Left hydrocele	Tumour/y	Yes, previously	Not done	Histology		
Bladder lesion at cystoscopy	Left orchiectomy	Cure									
Wedel & Jess/1991	Nigeria	1	13	*S.h.?*	Testicular granuloma	Tumour/y	No	Not done	Histology	Excision of the granuloma by saving the testicle, PZQ	?
Ahmed *et al*./2022	Yemen	1	15	*S.h.*	Acute epididimo-orchitis, testicular torsion	Testicular torsion/y	No	Not done	Histology	Orchiectomy followed by PZQ 400 mg	On observation
Ihekwaba, 1992	Nigeria	1	16	*S.h.*	Granulomatous testicular mass	Tumour/y	No	Not done	Histology	Orchiectomy followed by Niridazole 25 mg kg^−1^ d^−1^ 7 days	Not reported
Badmus *et al*./2012	Nigeria	1	16	*S.h.*	Granulomatous testicular nodules and mass	Tumour/y	No	Not done	Histology	Orchiectomy	Follow-up for 26 months.
Dauda/2006	Nigeria	1	16	*S.h.*	Granulomatous testicular mass	Tumour/y	No	Neg	Histology	Orchiectomy	Not reported
Chaves & Figueiredo/1965	Brazil	1	17	*S.m.*	Whitish nodules and papillomas, spread on scrotal skin	STD/y	No	Not done	Histology	Antimon	Healed
Walther, 1979	Puerto Rico	1	17	*S.m.*	Nodules and papillomas on scrotal skin	STD/y	No	Not done	Histology	None	Spontaneously healed
Richter, unpublished	Sierra Leone	1	17	*S.m.*	Hydrocele	Screening/no	No	Neg	Stool microscopy	PZQ 40 mg kg^−1^, 3 doses	Only partial recovery after PZQ, recovery after surgery
Richter, unpublished	Guinée Conakry	1	17	*S.h.*	Hydrocele	Screening/no	No	Neg	Serology	PZQ 40 mg kg^−1^, 3 doses	Lost to follow-up

Adeyemi Doro *et al*. (1979); Ahmed et al. (2022); Aminu et al. (2023); Badmus et al. (2012); Chaves and Figueiredo (1965); Dauda (2006); Ekenze et al. (2015); Eltayeb *et al*. (1969); Gelfand *et al*. (1940); Githae (1992); Ihekwaba *et al*. (1992); Joshi (1967); Lukacz *et al*. (1989); Madden (1911); Monnet et al. (1972); Oguntunde et al. (2020); Pawel et al. (2008); Rambau *et al*. (2011); Richter et al. (2002); Walther (1979); Wedel & Jess (1991); Ze Ondo *et al*. (2014).

## Literature search

Literature search was performed in several data bases including PubMed, Medline, Cochrane, Google scholar and Embase with the search terms ‘Male genital schistosomiasis AND child, children’ and ‘Male genital schistosomiasis AND adolescent/adolescents’. The selection process of eligible publications is shown in.

## Discussion

The importance of genital manifestations in women and men has been increasingly acknowledged during the last 30 years (Richter et al., 1995, 1996, 2008; Kjetland et al., 1996; Helling Giese et al., 1996a; Helling-Giese et al., 1996b; Leutscher et al. 2005; Schanz et al., 2010; Christinet et al., 2016; Kayuni et al., 2018; World Health Organization, 2020; Bustinduy et al., 2022; Fusco et al., 2022; Kutz et al., 2023; Shanaube et al., 2024).

However, knowledge on the importance of genital schistosomiasis in children and adolescents is scarce although genital schistosomiasis in a young boy has been described for the first time by Madden already in 1911. (Madden, 1911; Feldmeier et al., 1995; Bustinduy et al., 2022; Aribodor et al., 2024). There has never been a systematic investigation on the importance of genital manifestations at a young age. We came across four cases; two of them observed during health screening of unaccompanied minor refugees to Germany. To our knowledge, our case 1 is the youngest case of genital schistosomiasis age ever reported in the literature (Richter et al., 2002). Another case of penile schistosomiasis has been reported to one of our authors (B. Quire personal communication to AL Bustinduy). Surprisingly, besides our cases, we have found only other 23 cases published worldwide since 1911. Other publications on patient series with MGS have been published but it is not possible to understand from the publications whether or not minors included in the cohorts had MGS manifestations (Alves et al., 1955; Gelfand *et al*. 1940; De Souza *et al*. 2004; Aminu *et al*. 2023; Mohammed et al., 2007; Ramarokoto et al., 2008; Msyamboza et al., 2010; Percheron et al., 2024). The relative scarcity of cases published in the literature points at underreporting. One possible reason is that only a minority of patients present with haematuria. In our case series, only 3/27 (11.11%) cases reported haematuria. Another indicative sign of MGS, i.e. haematospermia and changes of the consistence of ejaculate cannot be investigated before puberty (Corachan et al., 1994; McKenna et al., 1997; van Delft et al., 2007). A third reason is possibly that MGS may occur without the presence of schistosome ova in urine (Richter et al., 2002; van Delft et al., 2007). A fourth reason may possibly be that the condition is not well known to urologists. 24/27 (88.89%) cases of our series were diagnosed by accident when another cause was suspected such as a malignancy (see Table 1). Even in endemic countries urologists usually do not connect genital problems with schistosomiasis and even less so, when it occurs in children or adolescents. In endemic countries, hydrocele is most frequently ascribed to filariasis. Interestingly, contrary to current notions, in an Egyptian and In a Sudanese case series on histopathological samples collected during scrotal surgery, schistosomiasis was the cause of scrotal swellings more frequently than filariasis (Abdel Wahab et al., 1981; Malik et al. 1982). A fifth possible biasing cause is the fact that the patients and their parents are ashamed of their condition which, on the other hand, appears to be painless and not immediately threatening. In fact, in our cases with hydrocele, the patients had never reported spontaneously their ailment to the medical staff at the first visit and mostly asked later to be seen by a male doctor.

In adults, genital involvement is particularly frequent condition in infections by *S. haematobium* and to a lesser extent *S. intercalatum* (Corachan et al., 1987; Picaud et al., 1990; Jusot et al., 1997; Leutscher et al., 2000; van Delft et al., 2007; Ramarokoto et al., 2008; Kayuni et al., 2018) but genital schistosomiasis has been reported to occur also in *S. mansoni, S. japonicum* and *S. matthei* infections (Kayuni et al.^2024^; Steinberger et al., 1975; Armbrust, 1951; Steinberger et al., 1975; Bambirra et al., 1986; Lee et al., 2000; Shekhar et al., 2000; de Souza Alves et al. 2004; Cassio Saito et al., 2004; Neto et al., 2004; Lopes et al., 2007a, 2007b; Ricosse et al., 1980; Schwartz et al., 2002).

Another particular genital manifestation of schistosomiasis had been addressed by Brandt *et al*. (2003): severe hepatosplenic schistosomiasis may be associated with hypogonadism and, after porto-systemic collaterals have evolved or been surgically created, with varicocele. Specifically, hypogonadism and retarded sexual development as a manifestation of schistosomiasis which is not systematically explored (Brandt et al., 2003; Jatsa et el. 2022).

The frequency of the diagnosis of genital schistosomiasis depends also on the diagnostic method. In autopsy digest methods are far more sensitive than histology for detecting schistosome ova (Edington et al., 1975). For detecting scrotal abnormalities scrotal ultrasonography has to be done. In screening programmes, a subject who does not think to have involvement of the scrotal organs is not likely to easily accept scrotal ultrasonography. Examination by transrectal ultrasonography is more sensitive for detecting prostatic lesions than transabdominal ultrasonography (Vilana et al., 1997; Al Saeed et al. 2003). On the other hand, transrectal ultrasound is usually not applied in children, more time consuming than transabdominal ultrasound alone and is probably not that easily accepted in a field context where privacy is not that easily felt to be warranted.

## Conclusions

1. Male genital schistosomiasis is a manifestation of schistosomiasis, which is neglected specially when occurring in children or adolescents.
2. Paediatricians and urologists should be aware of this condition to avoid stigmatization or unnecessary surgery.
3. Shame and fear of stigmatization may play a role of the particular neglect of this condition, also, because on the other hand, MGS in this age appears not to be particularly painful or threatening to the young patients.
4. Scrotal ultrasonography may require extensive and careful explanation to the patients and their parents if these methods are planned to be integrated into a screening programme.
5. Praziquantel treatment, when given before surgery appears to be efficient in most cases although, in some cases, surgery may be required.
6. The occurrence of MGS already in early childhood underscores the concept to treat schistosomiasis at an early age to prevent unnecessary complications which untreated may require surgery or may become no more reversible (Bustinduy et al., 2017).
